# Supplementary material for: Risk Factors for Mortality in Adult COVID-19 Patients Who Develop Bloodstream Infections Mostly Caused by Antimicrobial-Resistant Organisms: Analysis at a Large Teaching Hospital in Italy
Source: J Clin Med. 2021 Apr 17;10(8):1752. doi: 10.3390/jcm10081752 (PMC8073579; doi:10.3390/jcm10081752)
Supplement: Supplementary file 1 [file jcm-10-01752-s001.zip › jcm-1158044-supplementary.pdf]

Table S1. Summary of microbiological characteristics of bloodstream infection episodes with or without an identified pulmonary source in 46 patients studied

Patient identifier	Episode characteristics		Episode-related pulmonary source characteristics	
	Episode identifier	Microbial species (antimicrobial resistance determinant identified)	Episode identifier	Microbial species (antimicrobial resistance determinant identified)
1	1.1	*Enterococcus faecalis* + *Proteus mirabilis*		
2	2.1	*Escherichia coli* (*bla*$_{\text{CTX-M-15}}$)		
3	3.1	*Staphylococcus aureus* (*mecA*)		
4	4.1	*Staphylococcus aureus* (*mecA*)		
5	5.1	*Klebsiella pneumoniae* (*bla*$_{\text{CTX-M-15}}$)		
6	6.1	*Enterococcus faecalis* + *Klebsiella pneumoniae* (*bla*$_{\text{KPC}}$)	6.1	*Klebsiella pneumoniae* (*bla*$_{\text{KPC}}$)
7	7.1	*Staphylococcus aureus*		
8	8.1	*Enterococcus faecium*		
9	9.1	*Staphylococcus aureus* (*mecA*)		
10	10.1	*Pseudomonas aeruginosa*	10.1	*Pseudomonas aeruginosa*
11	11.1	*Escherichia coli*		
12	12.1	*Candida parapsilosis*		
13	13.1	*Staphylococcus epidermidis*		
14	14.1	*Enterococcus faecalis*		
15	15.1	*Enterococcus faecalis* + *Streptococcus oralis*, *Candida krusei*		
16	16.1	*Proteus mirabilis*		
17	17.1	*Klebsiella pneumoniae* (*bla*$_{\text{KPC}}$)	17.1	*Klebsiella pneumoniae* (*bla*$_{\text{KPC}}$)
18	18.1	*Staphylococcus aureus* (*mecA*)		
19	19.1	*Staphylococcus aureus* (*mecA*)	19.1	*Acinetobacter baumannii* (*bla*$_{\text{OXA-23}}$) + *Pseudomonas aeruginosa* + *Staphylococcus aureus* (*mecA*)
20	20.1	*Candida albicans* + *Candida glabrata*		
21	21.1	*Staphylococcus aureus* + *Streptococcus anginosus* + *Streptococcus oralis*	21.1	*Staphylococcus aureus*
	21.2	*Pseudomonas aeruginosa*		
22	22.1	*Staphylococcus epidermidis*		
23	23.1	*Staphylococcus aureus* (*mecA*)		
24	24.1	*Staphylococcus aureus* (*mecA*)		
25	25.1	*Bacteroides fragilis*		
26	26.1	*Enterococcus faecalis*		
27	27.1	*Enterococcus faecalis*		
28	28.1	*Pseudomonas aeruginosa*	28.1	*Pseudomonas aeruginosa*
	28.2	*Staphylococcus aureus* (*mecA*)	28.2	*Staphylococcus aureus* (*mecA*)
29	29.1	*Klebsiella pneumoniae* + *Staphylococcus aureus*	29.1	*Klebsiella pneumoniae* + *Staphylococcus aureus*
30	30.1	*Streptococcus pneumoniae*	30.1	*Streptococcus pneumoniae*
	30.2	*Candida albicans*		
31	31.1	*Staphylococcus aureus* (*mecA*)		
	31.2	*Morganella morganii*		
	31.3	*Candida glabrata*		
	31.4	*Acinetobacter baumanni* (*bla*$_{\text{OXA-23}}$) + *Candida glabrata* (mutated *FKS*)		
32	32.1	*Enterococcus faecium* (*vanA*)	32.1	*Enterococcus faecium* (*vanA*)
33	33.1	*Enterococcus faecalis* + *Staphylococcus aureus* (*mecA*)		
34	34.1	*Staphylococcus aureus* (*mecA*)	34.1	*Klebsiella pneumoniae* + *Staphylococcus aureus* (*mecA*)
	34.2	*Stenotrophomonas maltophilia*		
35	35.1	*Staphylococcus aureus*	35.1	*Staphylococcus aureus*
36	36.1	*Staphylococcus aureus* (*mecA*)	36.1	*Staphylococcus aureus* (*mecA*)
37	37.1	*Streptococcus pneumoniae*	37.1	*Citrobacter koseri* + *Streptococcus pneumoniae*
38	38.1	*Pseudomonas aeruginosa*	38.1	*Pseudomonas aeruginosa*
	38.2	*Enterococcus faecalis*		
39	39.1	*Enterococcus faecalis*		
40	40.1	*Staphylococcus aureus* (*mecA*)	40.1	*Staphylococcus aureus* (*mecA*)
41	41.1	*Staphylococcus aureus* (*mecA*)		
	41.2	*Candida albicans*		
42	42.1	*Candida parapsilosis*		
	42.2	*Klebsiella pneumoniae* (*bla*$_{\text{KPC}}$)	42.2	*Klebsiella pneumoniae* (*bla*$_{\text{KPC}}$) + *Pseudomonas aeruginosa*
43	43.1	*Enterococcus faecalis*		
44	44.1	*Acinetobacter baumannii* (*bla*$_{\text{OXA-23}}$)	44.1	*Acinetobacter baumannii* (*bla*$_{\text{OXA-23}}$) + *Klebsiella pneumoniae* (*bla*$_{\text{KPC}}$)
45	45.1	*Pseudomonas aeruginosa*	45.1	*Pseudomonas aeruginosa*
	45.2	*Bacteroides fragilis* + *Proteus mirabilis*		
46	46.1	*Staphylococcus aureus* (*mecA*)	46.1	*Staphylococcus aureus* (*mecA*) + *Stenotrophomonas maltophilia*
	46.2	*Pseudomonas aeruginosa*		

Table S2. Etiology, type and source of 58 bloodstream infection episodes included in the study

Microbial species (no. of isolates)	No. (%) of episodes			No. (%) of episodes with a respiratory source (n = 20)
	All (n = 58)	Monomicrobial (n = 49)	Polymicrobial (n = 9)	
Gram-positive organisms (38)[a]				
Staphylococcus aureus (19)	19 (32.8)	16 (32.7)	3 (33.3)	9 (45.0)
Methicillin-resistant *S. aureus* (15)	15 (25.9)	14 (28.6)	1 (11.1)	
Coagulase-negative *Staphylococcus* species (2)	2 (3.5)	2 (4.1)		
Enterococcus faecalis (10)	10 (17.2)	6 (12.2)	4 (44.4)	
Enterococcus faecium (2)	2 (3.5)	2 (4.1)		1 (5.0)
Vancomycin-resistant *E. faecium* (1)	1 (1.7)	1 (2.0)		1 (5.0)
Streptococcus species (5)	4 (6.9)	2 (4.1)	2 (22.2)[b]	2 (10.0)
Gram-negative organisms (22)[a]				
Enterobacterales (12)	12 (20.7)	8 (16.3)	4 (44.4)	4 (20.0)
Third-generation cephalosporin-resistant Enterobacterales (2)	2 (3.5)	2 (4.1)		
Carbapenem-resistant Enterobacterales (3)	3 (5.2)	2 (4.1)	1 (11.1)	
Pseudomonas aeruginosa (6)	6 (10.3)	6 (12.2)		4 (20.0)
Antipseudomonal cephalosporin-resistant *P. aeruginosa* (1)	1 (1.7)	1 (2.0)		1 (5.0)
Antipseudomonal carbapenem-resistant *P. aeruginosa* (2)	2 (3.5)	2 (4.1)		
Acinetobacter baumannii (2)	2 (3.4)	1 (2.0)	1 (11.1)	1 (5.0)
Extensively drug-resistant carbapenem-resistant *A. baumannii* (2)	2 (3.4)	1 (2.0)	1 (11.1)	
Bacteroides fragilis (2)	2 (3.4)	1 (2.0)	1 (11.1)	
Yeast organisms (9)[a]				
Candida species (9)	8 (13.8)	5 (10.2)	3 (33.3)[b]	
Echinocandin-resistant *Candida glabrata* (1)	1 (1.7)		1 (11.1)	

[a] Detection of antimicrobial-resistance associated genes (within parentheses) was used to confirm antimicrobial-resistant phenotypes for methicillin-resistant *S. aureus* (*mecA*), vancomycin-resistant *E. faecium* (*vanA*), third-generation cephalosporin-resistant Enterobacterales ($bla_{CTX\text{-}M\text{-}15}$), carbapenem-resistant Enterobacterales (bla_{KPC}), carbapenem-resistant *A. baumannii* ($bla_{OXA\text{-}23}$), and echinocandin-resistant *C. glabrata* (mutated *FKS*) using previously described methods.[19,20]

[b] Two isolates (1 *Streptococcus* species and 1 *Candida* species) from two polymicrobial episodes for which another 1 *Streptococcus* species and 1 *Candida* species were respectively isolated are not listed. Accordingly, total number of isolates from the polymicrobial episodes was 20 and not 18 (as opportunely reported).

Table S3. Details about the empirical antimicrobial therapy in COVID-19 patients with or without bloodstream infection (BSI)[a]

Antimicrobial drug used	Patients with BSI (n = 46) who received			Patients without BSI (n = 50) who received
	Any (appropriate or inappropriate) therapy	Inappropriate therapy	Reason(s) for inappropriate therapy	Any therapy
Amoxicillin/clavulanic acid	1	–	–	0
Carbapenems				
Alone	4	4	1 *E. faecium*, 1 *bla*$_{KPC}$ positive *K. pneumoniae*, 1 MRSA, 1 carbapenem-resistant *P. aeruginosa*	1
Plus daptomycin	1	–	–	0
Plus linezolid	7	–	–	0
Plus vancomycin	2	–	–	1
Ceftazidime	1	–	–	0
Ceftriaxone				
Alone	0	–	–	4
Plus azithromycin	0	–	–	15
Piperacillin/tazobactam				
Alone	3	2	1 *bla*$_{CTX-M-15}$ positive *E. coli*, 1 MRSA	6
Plus linezolid	5	1	1 *bla*$_{OXA-23}$ positive *A. baumannii*	2
Plus vancomycin	6	–	–	1
Vancomycin	1	–	–	0
Any drug	31	7	See above reasons	30

[a] Fifty patients were random selected from those included in the study (*n* = 215) and used as a comparator group. Fifteen of 46 patients with BSI and 20 of 50 patients without BSI were untreated at the time of first blood culture collection.

Table S4. Predictive factors for survival in COVID-19 patients with bloodstream infection

	Kaplan-Meier analysis			Univariable Cox regression analysis		Multivariable Cox regression analysis	
	No. of death (*n* = 20)	Percent of survival[a]	*P* value	Hazard ratio (95% CI)	*P* value	Hazard ratio (95% CI)	*P* value
Age, years							
≤75	10	50		1.00 (reference)		1.00 (reference)	
>75	10	32	0.02	2.72 (1.12–6.59)	0.02	2.97 (1.15–7.68)	0.02
Male sex							
No	7	0		1.00 (reference)		–	
Yes	13	57	0.08	0.44 (0.17–1.14)	0.09	–	–
Charlson comorbidities index score							
≤3	11	60		1.00 (reference)		–	
>3	9	27	0.12	1.98 (0.81–4.84)	0.13	–	–
Hypertension							
No	10	35		1.00 (reference)		–	
Yes	10	54	0.71	1.17 (0.48–2.83)	0.71	–	–
COVID-19 severity status							
Moderate	4	74		1.00 (reference)			
Severe	11	55		0.89 (0.28–2.86)	0.85	–	
Critical	5	38	0.68	1.37 (0.35–5.26)	0.64	–	–
SOFA score							
≤2	3	80		1.00 (reference)		–	
>2	17	48	0.37	1.71 (0.50–5.87)	0.38	–	–
C-reactive protein, mg/L							
≤90	4	52		1.00 (reference)		1.00 (reference)	
>90	16	39	0.02	3.41 (1.12–10.32)	0.03	2.44 (0.72–8.25)	0.15
Procalcitonin, pg/mL							
≤2	13	58		1.00 (reference)		–	
>2	7	20	0.06	2.33 (0.91–5.95)	0.08	–	–
Interleukin 6, pg/mL							
≤200	12	37		1.00 (reference)		–	
>200	8	52	0.80	0.89 (0.36–2.19)	0.80	–	–
Septic shock							
No	8	74		1.00 (reference)		1.00 (reference)	
Yes	12	0	<0.001	5.10 (2.07–12.61)	<0.001	6.55 (2.36–18.23)	<0.001
Respiratory source of infection							
No	13	39		1.00 (reference)		–	
Yes	7	51	0.07	0.43 (0.17–1.12)	0.09	–	–
Recurrent infection							
No	19	38		1.00 (reference)		–	
Yes	1	80	0.17	0.27 (0.03–2.03)	0.20	–	–
ICU-acquired infection							
No	8	47		1.00 (reference)		–	
Yes	12	46	0.57	0.76 (0.30–1.93)	0.57	–	–
Time to infection onset, days							
>3	13	50		1.00 (reference)		1.00 (reference)	
≤3	7	36	0.001	4.96 (1.73–14.24)	0.003	4.68 (1.40–15.63)	0.01
Inappropriate empirical antimicrobial therapy							
No	12	33		1.00 (reference)		1.00 (reference)	
Yes	8	59	0.66	0.82 (0.33–2.01)	0.67	1.30 (0.42–4.03)	0.65
ICU admission							
No	3	69		1.00 (reference)		–	
Yes	17	43	0.95	0.96 (0.27–3.38)	0.95	–	–
GP bacterial infection							
No	6	34		1.00 (reference)		–	
Yes	14	55	0.56	0.75 (0.28–2.00)	0.57	–	–
GN bacterial infection							
No	10	60		1.00 (reference)		–	
Yes	10	35	0.50	1.34 (0.55–3.27)	0.51	–	–
Yeast infection							
No	16	55		1.00 (reference)		–	
Yes	4	0	0.78	0.78 (0.38–3.50)	0.78	–	–
Antimicrobial-resistant infection							
No	9	57		1.00 (reference)		1.00 (reference)	
Yes	11	35	0.72	1.17 (0.48–2.83)	0.72	0.80 (0.30–2.14)	0.66

COVID-19, coronavirus disease 2019; SOFA, sequential organ failure assessment; ICU, intensive care unit; GP, Gram-positive; GN, Gram-negative. *P* values of <0.05 are considered to be statistically significant.

[a] Measured during stay in or at discharge from the hospital for 46 COVID-19 patients who developed bloodstream infection.
